# Tm^3+^/Ho^3+^ profiled co-doped core area optical fiber for emission in the range of 1.6–2.1 µm

**DOI:** 10.1038/s41598-023-41097-2

**Published:** 2023-08-26

**Authors:** Piotr Miluski, Krzysztof Markowski, Marcin Kochanowicz, Marek Łodziński, Jacek Żmojda, Wojciech A. Pisarski, Joanna Pisarska, Marta Kuwik, Magdalena Leśniak, Dominik Dorosz, Tomasz Ragiń, Valiantsin Askirka, Jan Dorosz

**Affiliations:** 1 Faculty of Electrical Engineering, Bialystok University of Technology, Wiejska 45D Street, 15-351 Bialystok, Poland; 2 Faculty of Geology, Geophysics and Environment Protection, AGH University of Science and Technology, 30 Mickiewicza Av., 30-059 Krakow, Poland; 3 Institute of Chemistry, University of Silesia, 9 Szkolna Street, 40-007 Katowice, Poland; 4 Faculty of Materials Science and Ceramics, AGH University of Science and Technology, 30 Mickiewicza Av, 30-059 Krakow, Poland

**Keywords:** Optics and photonics, Applied optics, Lasers, LEDs and light sources

## Abstract

Double-clad optical fiber with a multi-ring core profile doped with thulium and holmium fabricated by Modified Chemical Vapor Deposition Chelate Doping Technology (MCVD-CDT) is presented. The measured Tm_2_O_3_ and Ho_2_O_3_ complexes’ weight concentrations were 0.5% and 0.2% respectively. Numerical analyses show weakly guiding conditions and 42.2 µm of MFD LP_01_ at 2000 nm. The low NA numerical aperture (NA = 0.054) was obtained for the 20/250 µm core/cladding ratio optical fiber construction. The emission spectra in the range of 1.6–2.1 µm vs. the fiber length are presented. The full width at half maximum (FWHM) decreases from 318 to 270 nm for fiber lengths from 2 to 10 m. The presented fiber design is of interest for the development of new construction of optical fibers operating in the eye-safe spectral range.

## Introduction

Currently, there is observed fast progress in the field of optical radiation sources based on fiber optic structures. Well-known of them are fiber lasers and Amplified Spontaneous Emission (ASE) sources. Among the different spectral ranges of optical radiation, the eye-safe range (above 1.4 µm) is especially attractive for numerous applications in medicine, meteorology, military, manufacturing, and detection systems^1–7^. The lasers operating in the spectral range of 1.7–2.1 µm are used in precise material processing (including cutting, drilling, engraving, and surface modification), medical applications (surgery), remote sensing (atmospheric monitoring), LIDAR (aerial mapping), Research and Development (spectroscopy, nonlinear optics, and quantum optics)^8–16^. Broadband ASE sources operating in the Near-Infrared (NIR) are commonly used for optical coherence tomography (OCT) imaging and spectroscopy^17^. The generation of radiation in the structure of optical fiber arises as a result of the emission of radiation by ions of rare earth elements. For the discussed spectral range, these are usually Tm^3 + ^and Ho^3 + ^ions. They allow for obtaining a wide emission profile in the range of 1.7–2.1 µm (7.21). Moreover, it is possible to change the emission profile through the use of phenomena like co-emission, energy transfer, and cross-relaxation^18^. Sometimes doping with Yb^3 +^ ions is also used, which acts as a sensitizer, enabling the use of popular laser diodes in the 980 nm range to excite holmium. The transitions of Tm^3 + ^: ^3^F_4_ →  ^3^H_6_ (around 1.8 µm) and Ho^3 + ^: ^5^I_7_ →  ^5^I_8_ (around 2.0 µm) are responsible for the emission within investigated spectral region. Typically, in the Tm^3 +^ -Ho^3 + ^ co-doped system, the Ho^3 + ^ions are excited using energy transfer from Tm^3 +^ (exc. at 800 nm) or Yb^3 + ^ (exc. at 980 nm) sensitizers^19–22^. An interesting research aspect is optical fibers with side emission and those in which radiation is generated using up-conversion phenomena. This allows the emission of a shorter wavelength spectrum than the excitation radiation using the multiphoton absorption mechanism^23,24^. The commonly used technology for fabrication active (doped with lanthanides ions) optical fibers is modified chemical vapor deposition (MCVD) with solution doping technique (SDT) and chelate doping technology (CDT) 28. In fact, some challenges and limitations of SDT (dopant distribution stability, small core diameter, and multi-stage process of preform production) cause the CDT technology currently being intensively developed for active fibers production. MCVD-CDT enables the production of preforms with a large core diameter with better control of process parameters and high repeatability^25–28^. The development of new single-mode and high-power broadband sources is still very attractive for industry and scientific research. Among the optical fiber constructions, the single-mode fibers with a wide mode area (Large Mode Area fibers) undoubtedly attract the most attention^29–31^. Their propagation properties (lower level of optical power density, reduction of photodarkening effect, and well-defined beam shape with a wide mode field). The key parameter is the mode field width in such fibers (Mode Field Diameter, MFD), which, unlike classical fiber designs, is significantly larger and leads to the fact that the effective mode area can be even 100 times bigger (up to 1000 µm^2^) for LMA fibers^32–35^. Commercial LMA fibers have a mode field width of 22.4 µm (LMA-TDF-25P/250-HE, Nufern) or 21.5 µm (LMA-YDF-30/250-HI-M +, Coherent). The results of scientific research also indicate the possibility of obtaining a wide mode field (MFD = 35 µm) for LMA fiber with a small numerical aperture NA = 0.0281^39^. That’s why an important research aspect is the development of new active LMA optical fibers with a possibly wide modal field. Such a profile can be obtained by a multi-ring design of the refractive profile. Moreover, this type of spatial distribution of the dopant makes it possible to optimize the luminescence profile through the phenomena of co-emission and energy transfer of rare earth elements^36–43^. In the described case, alternating layers of multi-ring Tm^3 +^ /Tm^3 + ^Ho^3 + ^were used for this purpose. The aim was to obtain a fiber with a wide mode field and a flat profile of broadband emission obtained as a result of radiation generation (Tm^3 +^ /Tm^3 + ^Ho^3 +^) in the structure of the developed fiber. The low numerical aperture (low ∆n) in multiple active layer construction of fibre core ensures weakly guiding of the fundamental mode in the large core (LMA). The composition of Tm^3 +^ and Tm^3 + ^Ho^3 +^ layers was used to obtain broadband and smooth flat profile of the spectrum in the fabricated fiber.

## Methods

The numerical analysis of optical properties for the proposed optical fiber design was performed using the mode solver of RP Fibre Power software. Additionally, the spectral properties (ASE forward and backward forming) vs. the fiber length were simulated. The effective index, number of modes, fraction of power in the core, and luminescence spectrum profile were investigated for steady-state transmission. The simulations used measured refractive index and dopants’ concentration profile for the fiber. The calculations and analysis were performed in the range of 1400–2000 nm. The mode field diameter was determined according to reduction factor 1/e. The proposed novel multi-ring structure of the refractive index and dopants’ distribution profile requires a multi-step deposition process. The MCVD-CDT technology is a reliable and efficient way to fabricate large core diameter optical fibers. It is well known that the incorporation of a high concentration of lanthanides into the silica host is limited due to the solubility and host modification. Phase separation is a common challenge in high-concentration laser construction. It is well known that rare-earth ions solubility can be increased by introducing modifiers (aluminum and/or phosphorus) but one can expect an increase in the refractive index delta (∆n) in the profile. While the LMA fibers require a relatively low refractive index delta. On the other hand, the intensity of the luminesce depends on the dopants’ concentrations. As a compromise, we decided to fabricate multi-ring core optical fiber with low alumina concentration. The preform was fabricated in the Bialystok University of Technology using the Optacore Modified Chemical Vapor Deposition Chelate Doping Technology (MCVD-CDT) system. Tm(tmhd)_3_ and Ho(tmhd)_3_, complexes were used as lanthanides precursors with a helium carrier gas. The oxygen lines were used for silicon tetrachloride delivery to the ultra-pure Herause F300 tube (28/24 mm outer/inner diameter). The closed-loop temperature control system with a pyrometer (burner type) and mass flow controllers was used for the deposition rates and hot zone control. The refractive index profiles were measured using the P104 preform analyzer (632.8 nm). The designed double-clad structure of optical fiber (core to cladding ratio 20/250 µm) required additional stretching and jacketing process using the MCVD system. The outer cladding was fabricated during the drawing process (UV-cured polymer cladding n = 1.375). This process was performed using Control Interface drawing tower (Centorr furnace @ 2050 °C). The luminescence spectra of the preform and fibers (OSA) were measured using laser diode 796 nm (CW, regulated in the range 1–30 W) and Optical Spectrum Analyzer (1.6–2.1 µm) Yokogawa AQ6375B. The concentration of lanthanides and glass-forming elements in the cross-section of optical preforms was measured using a scanning electron microscope equipped with EDS (EDAX) detector. The JEOL SuperProbe JXA-8230 was used with a voltage of 15 kV, beam current 20 nA, and resolution < 1 µm. The decay time was measured for the preform slide (2 mm thickness) for excitation of the all area of fibre core. Decay time measurements of Tm^3+^ and Ho^3+^ ions were carried out using the PTI QuantaMaster QM40 system, coupled with a tunable pulsed optical parametric oscillator (OPO), pumped by a third harmonic of an Nd:YAG laser (OpotekOpolette 355 LD). The laser system was equipped with a double 200 mm (focal length) monochromator, a multimode UV–VIS photomultiplier tube (PMT) (R928), and Hamamatsu H10330B-75 detectors controlled by a computer. Luminescence decay curves were recorded and stored by a PTI ASOC-10 [USB-2500] oscilloscope.

## Results and discussion

### Numerical analysis of optical fiber

**Figure 1:**
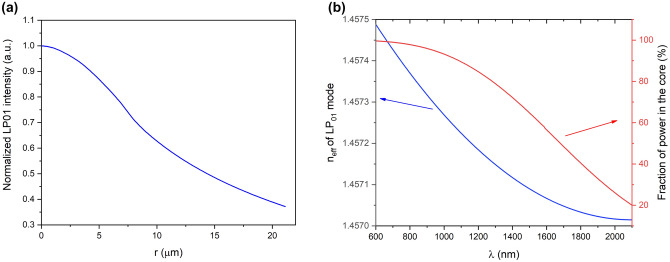
(**a**) LP_01_ mode profile (λ = 2000 nm), (**b**) effective index of the LP_01_ mode and the fraction of power in the core.

The main goal of the construction of multi-ring Tm^3+^/Tm^3+^Ho^3+^ fiber was to obtain a high effective mode area while maintaining a single-mode operation. The modal properties simulations were carried out for the measured refractive index profile (scaled) of the optical fiber preform refractive index profile (Fig. 4a). The number of propagated modes was calculated and the cut-off wavelength was estimated to λ_c_ = 780 nm. The profile of the mode is important in the LMA active fibers due to the fact that the optical beam propagates in the core and the cladding simultaneously. For that reason, the profile intensity was calculated and the Mode Field Diameter (MFD) 42.2 µm (at 1/e) of the fundamental mode LP_01_ intensity profile was obtained for λ = 2000 nm (Fig. 1a).

The effective refractive index of the developed fiber design varies in the range of 1.4575 to 1.4571 for 600 nm and 2100 nm respectively (Fig. 1b). Obtaining a large mode area propagation in fibers with a step refractive index profile can be achieved by increasing the core size. However, at the same time, this significantly reduces the numerical aperture to impractical values^37^. The low NA promotes the reduction of optical power in the fiber core and reduces the non-linear effects for high-power applications. However, significant losses can be introduced by optical fiber structure imperfections, cladding region attenuation, and fiber bending (coupling with cladding modes) when the NA is lower than 0.06^41^. Modal sensitivity and fundamental mode optical power of transfer to higher order modes is a common problem then. Therefore, the short-length rare-earths doped fibers used for lasers and amplifiers working with single-mode regime constructions often require additional cladding modes strippers (e.g. high refractive index coatings or fiber coiling) to obtain the best possible LP_01_ beam quality (M^2^ ≈ 1)^42^. The significant change in the fraction of power in the fiber core propagated can be noticed vs. the propagation wavelength and only 20% of optical power (weak guidance) will be propagated in the fiber core for 2100 nm (Fig. 1b).

**Figure 2:**
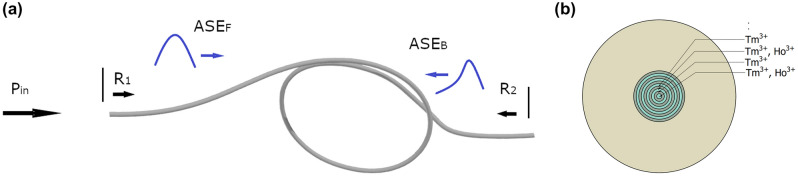
(**a**) The ASE generation schematic simulation, R_1_ and R_2_ reflection on the input and output face of the optical fibers respectively, ASE_F_ and ASF_B_—Amplified Spontaneous Emission in the forward and backward direction respectively, (**b**) cross-section optical fiber (doping structure).

**Figure 3:**
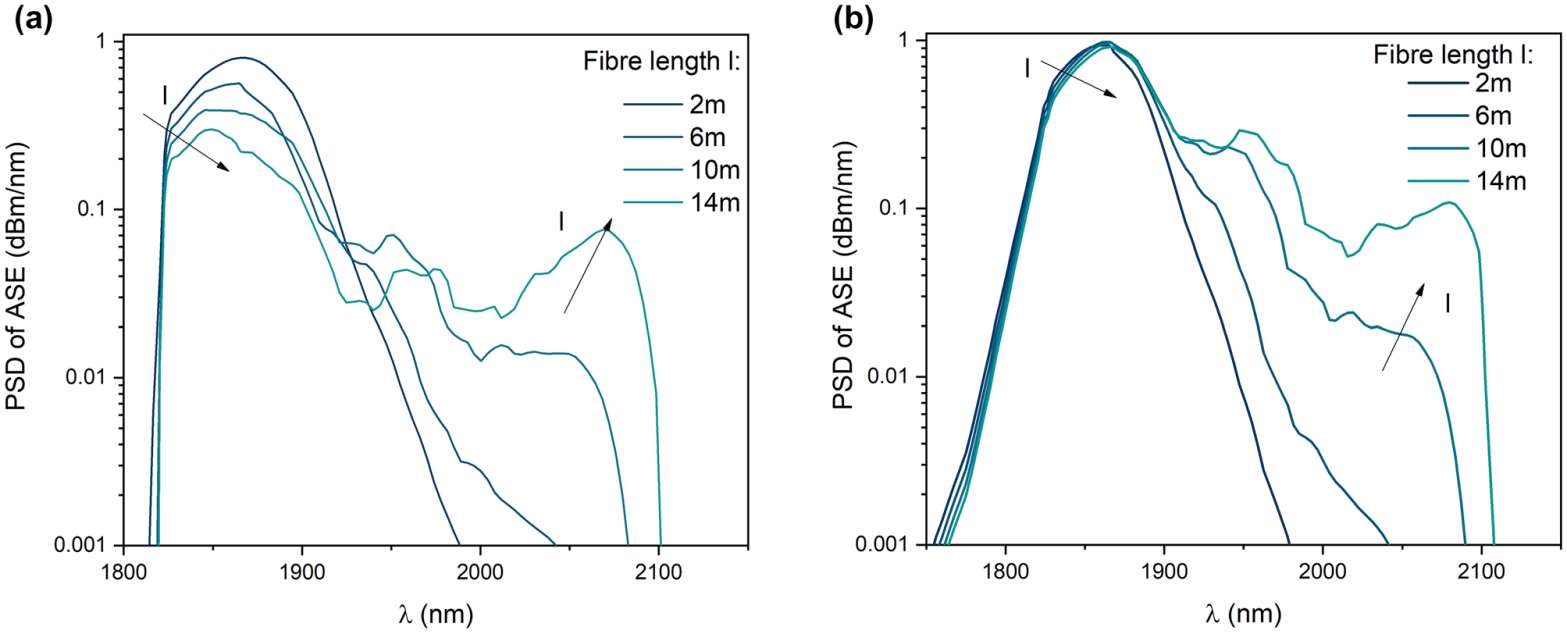
The Luminescence spectrum calculated for 796 nm single mode excitation for (**a**) forward and (**b**) backward directions.

**Figure 4:**
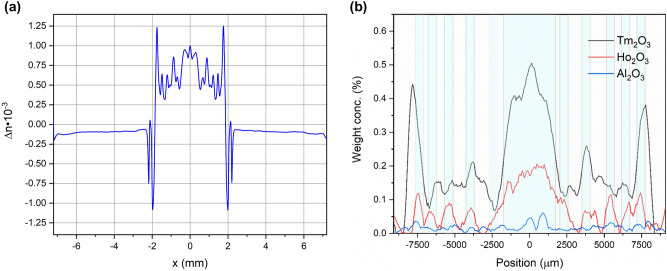
Fabricated preform characterization: (**a**) ∆n, refractive index profile (**b**) EPMA-EDS profile of Tm_2_O_3_, Ho_2_O_3_, Al_2_O_3_ weight concentration.

The ASE was simulated at single-end core fiber excitation (Fig. 2a). The Tm^3+^ and Ho^3+^ ions concentrations profiles were calculated using the results of energy-dispersive x-ray spectroscopy measurements (Fig. 4b). The spectral properties of the fiber simulations were limited to the assumption that the pump and the signal are propagating in the fiber core (the cladding pumping mechanism wasn’t investigated). The forward CW pump radiation at 796 nm, Gauss-profile shape, and pulse power 3W was analyzed. The single mode propagation for the ASE channel and silica/air interface reflections on both optical fiber ends were assumed. The Amplified Spontaneous Emission in the forward and backward direction (ASE_F_ and ASF_B_) was simulated for a fiber length range of 2–14 m (1000 calculation steps per fiber length, 5 nm per channel spectrum). The Power Spectral Density (PSD) of ASE signals in forward and backward directions were investigated in the range of 1550–2100 nm (Fig. 3a,b).

The normalized spectrum profile is significantly different in both ASE forming directions. It can be noticed that the holmium emission band (2000–2100 nm) associated with the transition ^5^I_7_ → ^5^I_8_ increase versus the fiber length. This phenomenon is related to the energy transfer mechanism Tm → Ho ions and is more intense in the spectrum of ASE backward directions (Fig. 3b). It is worth mentioning that, the method of combined generation of both ends can be used to obtain a more uniform emission profile.

### Fiber characterization

The preforms were analyzed in terms of refractive index profile and dopants’ concentration measurements. The refractive index profile as well as Tm_2_O_3_, Ho_2_O_3_, and Al_2_O_3_ concentration are presented in Fig. 4a,b. Analyzing the presented graphs, one can notice a correlation between the concentrations of lanthanides (thulium and holmium) and the increase in the refractive index. The influence of the doping concentration of lanthanides for refractive index value can be estimated using the following equation^40^:

1 $$\Delta n={10}^{-4}\cdot \left[67\cdot \left({C}_{\mathrm{RE}}\right)+22\cdot {C}_{\mathrm{Al}2\mathrm{O}3}\right],$$

where the ${C}_{RE}$, and ${C}_{\mathrm{Al}2\mathrm{O}3}$ refer to the molar % concentrations of rare-earths and aluminum oxides respectively.

**Figure 5:**
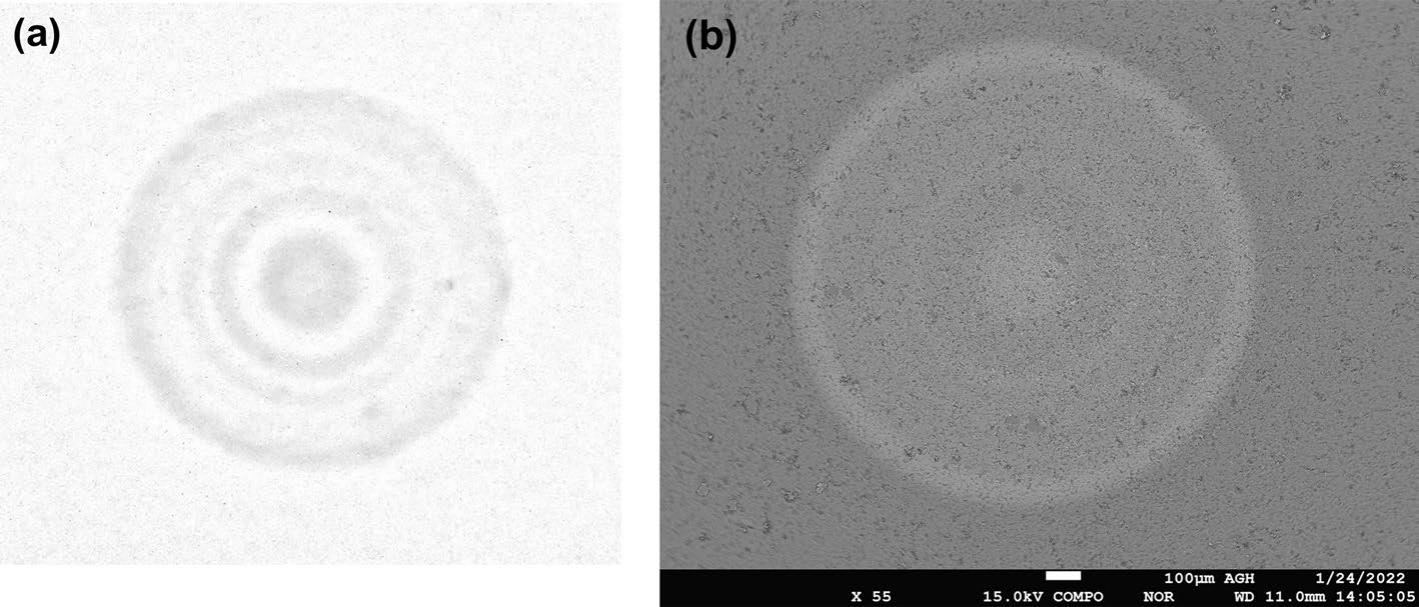
Fabricated preform characterization: (**a**) photo of preform core, (**b**) SEM image of the preform core.

In the case under consideration, the ∆n of the refractive index is relatively low (maximum 1.25·10^−3^). The deposition process was optimized to eliminate the center dip in the refractive index profile which is a common problem for MCVD technology^37^. This assures low numerical aperture value NA_core/cladd_ = 0.054 and promotes the single-mode operation. Low NA is important since it reduces the total number of guided modes for a large core diameter and provides reliable single-mode operation. Moreover, the doping profile should give preference to fundamental mode enhancement. The measured doping profile of the fiber core consists of 11 deposition stages. The Tm^3+^ is present in all of the ring layers while Ho^3+^ is present in 5 ring layers according to Fig. 2b. Both, the Tm^3+^ and Ho^3+^ dopants can be noticed in the electron probe microanalysis (EPMA) energy-dispersive X-ray spectroscopy (EDS) analysis result (Fig. 4b, Ho^3+^ was additionally marked as a blue background color). The maximum weight concentration of Tm_2_O_3_ and Ho_2_O_3_ complexes are 0.5% and 0.2% respectively while aluminum oxide concentration is below 0.05%. The low aluminum oxide concentration may lead to clustering phenomena with lifetime quenching^37^. The spatial profile of the doping is also visible in the photo (Fig. 5a) and the SEM image (Fig. 5b) of the preform. There are no clustering effects visible in the SEM image of the preform. The fact that aluminum has lower phonon energy than silica is particularly important in the construction of fiber lasers^43^. In our case, the fluorescence decay time was measured for Tm^3+^: ^3^F_4_ → ^3^H_6_ transition (excitation 796 nm, detection 1800 nm), and the single exponential decay time was 378 µs (adjusted R-Squared 0.994). The obtained result confirms that there is no significant clustering problem and is comparable with other reported fiber designs^41^.

The double-clad structure of optical fiber was drawn after the proper preform preparation. Obtaining the desired core/cladding ratio requires stretching the preform and jacketing (using F300 silica tube). The outer cladding was fabricated by the polymer cladding deposition during fiber drawing. As a result, the double-clad single-mode optical fiber (20 μm core and 250 μm cladding, NA_core/cladd_ = 0.054, NA_cladd/polymer_ = 0.482) was fabricated. The optical attenuation measured by the optical time-domain reflectometer at 1310 nm (2nd optical window) was estimated to c.a. 20 dB/km.

**Figure 6:**
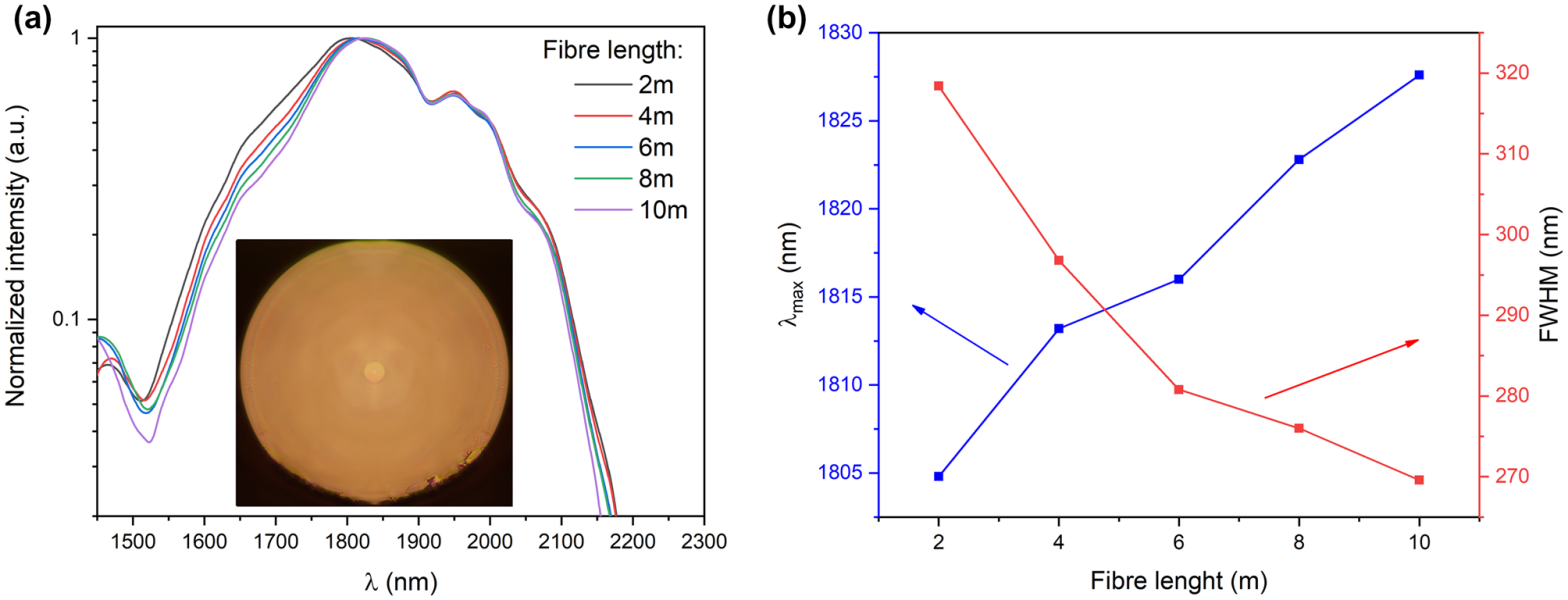
(**a**) The luminescence spectrum profile (inset–fiber photo), (**b**) the λ_max_ and FWHM vs the fiber length at exc. 796 nm.

The luminescence profiles vs. fiber length were measured using cladding pumping (excitation 796 nm) and the cut-back method. The luminescence was measured at the end of the fiber using an OSA instrument. The co-doping of the optical fiber profile results in eye-safe emission (in the range of 1.6–2.1 µm, 10 dB bandwidth). Moreover, the spectrum profile modification as a function of fiber length can be used for the optimization of its shape (Fig. 6a,b) since the slight red shift of the maximum emission profile is noticeable in the range of 1804 nm–1827 nm and full width at half maximum (FWHM) decreases from 318 to 270 nm for fiber length from 2 to 10 m. The emission profile changes due to the reabsorption process in the optical fiber structure (Tm^3+^: ^3^H_4_ → ^3^H_6_). In consequence, changes in the luminescence profile of thulium emission are noticeable. The results of numerical calculations of the luminescence spectra presented in Fig. 3a and b differ from those measured in the manufactured optical fiber (the measurement results show much smaller changes in the emission spectrum). The key parameter influencing the differences is the use in the calculations of the pumping mechanism only of the core area, unlike pumping with the jacket, which was used during measurements. This results in significantly faster absorption of the pump in the optical fiber structure. However, both the results of numerical calculations and measurements confirm the possibility of shaping the luminescence profile by selecting the length of the optical fiber. The Tm^3+^/Ho^3+^ co-doped fibers are often investigated as fiber laser constructions in the range of 1.7–1.8 µm^41^ and 2.0–2.1 µm obtaining tenths of watts output power^42^. The wideband emission-designed fibers were also investigated and demonstrated for ASE sources for flat spectral power density in the range from 1527 to 2171 nm (10 dB bandwidth)^43^. Our investigations show that wideband emission in the eye-safe spectral range can be obtained using the proposed design of LMA fiber.

## Conclusion

The silica Large Mode Area optical fiber (42.2 µm of MFD LP_01_ at 2000 nm) for emission in the eye-safe emission in the range of 1.6–2.1 µm was fabricated and characterized in terms of its luminescent properties. The normalized spectrum profile for both ASE forming directions was calculated. The holmium emission band (2000–2100 nm) associated with the transition ^5^I_7_ → ^5^I_8_ increase versus the fiber length due to the transfer mechanism Tm → Ho. The double-clad optical fiber (20 μm core and 250 μm cladding, NA_core/cladd_ = 0.054, NA_cladd/polymer_ = 0.482) with a multi-ring profile of thulium and holmium doping was fabricated using MCVD-CDT technology. The multi-ring profile with maximum Tm_2_O_3_ and Ho_2_O_3_ complexes weight concentrations 0.5% and 0.2% respectively were confirmed using SEM/EDS analysis. The full width at half maximum (FWHM) in the range from 318 to 270 nm was measured for fiber length from 2 to 10 m. The proposed fiber design can be used as a single-mode operation radiation source (in the range near 2 μm) in new photonics devices (environmental sensors, laser scanners, free space telecommunication, or scientific applications). The mode field width obtained in the proposed fiber construction is greater than that of commercially available active fibers, which will undoubtedly allow for further development and obtaining a wide mode profile in LMA fibers.

## Data Availability

The datasets used and/or analysed during the current study available from the corresponding author on reasonable request.

## References

[1] Sliney DH (1995). Laser safety. Lasers Surg. Med..

[2] Franks, J. K. What is eye safe? in (ed. Johnson, A. M.) 2–8 (1991). doi:10.1117/12.43840.

[3] Scholle K, Lamrini S, Koopmann P, Fuhrberg P, Scholle K (2010). 2 µm laser sources and their possible applications. Frontiers in Guided Wave Optics and Optoelectronics.

[4] Jackson SD (2012). Towards high-power mid-infrared emission from a fibre laser. Nat. Photon..

[5] Geng J, Wang Q, Lee Y, Jiang S (2014). Development of eye-safe fiber lasers near 2 μm. IEEE J. Sel. Top. Quantum Electron..

[6] Hoult, T. Lasers in the 2um SWIR spectral regime and their Applications. in *CLEO: 2015* ATu4M.1 (OSA, 2015). doi:10.1364/CLEO_AT.2015.ATu4M.1.

[7] Geng, J., Wang, Q. & Jiang, S. 2μm fiber laser sources and their applications. in (eds. Taylor, E. W. & Cardimona, D. A.) 816409 (2011). doi:10.1117/12.896259.

[8] Pierce MC, Jackson SD, Dickinson MR, King TA (1999). Laser-tissue interaction with a high-power 2-?m fiber laser: Preliminary studies with soft tissue. Lasers Surg. Med..

[9] Fried NM (2005). Thulium fiber laser lithotripsy: An in vitro analysis of stone fragmentation using a modulated 110-watt Thulium fiber laser at 1.94 µm. Lasers Surg. Med..

[10] Písařík M (2016). Thulium-doped fibre broadband source for spectral region near 2 micrometers. Opto-Electron. Rev..

[11] Theisen-Kunde D, Ott V, Brinkmann R, Keller R (2007). Potential of a new cw 2μm laser scalpel for laparoscopic surgery. Med. Laser Appl..

[12] Beyon JY (2007). High-energy 2 μm Doppler lidar for wind measurements. Opt. Eng..

[13] Henderson SW (1993). Coherent laser radar at 2 mu m using solid-state lasers. IEEE Trans. Geosci. Remote Sens..

[14] Voisiat, B. *et al.* Material processing with ultra-short pulse lasers working in 2μm wavelength range. in (eds. Roth, S., Nakata, Y., Neuenschwander, B. & Xu, X.) 935014 (2015). doi:10.1117/12.2078651.

[15] Shi W, Fang Q, Zhu X, Norwood RA, Peyghambarian N (2014). Fiber lasers and their applications [Invited]. Appl. Opt..

[16] Mingareev I (2012). Welding of polymers using a 2μm thulium fiber laser. Opt. Laser Technol..

[17] 1700 nm ASE light source and its application to mid-infrared spectroscopy | IEEE Conference Publication | IEEE Xplore. https://ieeexplore.ieee.org/abstract/document/6888126.

[18] Tanabe S (2002). Rare-earth-doped glasses for fiber amplifiers in broadband telecommunication. C. R. Chim..

[19] Zhang Q (2010). Infrared emission properties and energy transfer between Tm 3+ and Ho 3+ in lanthanum aluminum germanate glasses. J. Opt. Soc. Am. B.

[20] Li M (2013). ∼2 µm Luminescence and energy transfer characteristics in Tm3+/Ho3+co-doped silicate glass. J. Quant. Spectrosc. Radiat. Transf..

[21] Wang X (2015). Spectroscopic properties of Ho3+ and Al3+ co-doped silica glass for 2-μm laser materials. J. Lumin..

[22] Jackson SD (2009). The spectroscopic and energy transfer characteristics of the rare earth ions used for silicate glass fibre lasers operating in the shortwave infrared. Laser Photon. Rev..

[23] Mangini F (2020). Multiphoton-absorption-excited up-conversion luminescence in optical fiber. Phys. Rev. Appl..

[24] Ferraro M (2021). Femtosecond nonlinear losses in multimode optical fibers. Photon. Res..

[25] Schuster K (2014). Material and technology trends in fiber optics. Adv. Opt. Technol..

[26] Saha M, Pal A, Sen R, Saha M (2012). Vapor phase chelate delivery technique for fabrication of rare earth doped optical fiber. International Conference on Fibre Optics and Photonics TPo.12.

[27] Mat Sharif KA, Omar NYM, Zulkifli MI, Muhamad Yassin SZ, Abdul-Rashid HA (2020). Fabrication of alumina doped optical fiber preforms by MCVD-metal chelate doping method. Appl. Sci..

[28] Miluski P (2020). Eye safe emission in Tm3+/Ho3+ and Yb3+/Tm3+ co-doped optical fibers fabricated using MCVD-CDS system. Opt. Mater..

[29] Siegman AE, Fotakis C, Kalpouzos C, Papazoglou TG (1993). High-power laser beams: defining, measuring and optimizing transverse beam quality. 9th International Symposium on Gas Flow and Chemical Lasers.

[30] Paschotta R (2006). Beam quality deterioration of lasers caused by intracavity beam distortions. Opt. Express.

[31] Baggett JC, Monro TM, Furusawa K, Richardson DJ (2001). Comparative study of large-mode holey and conventional fibers. Opt. Lett..

[32] Jain D, Jung Y, Kim J, Sahu JK (2014). Robust single-mode all-solid multi-trench fiber with large effective mode area. Opt. Lett..

[33] Jain, D., Baskiotis, C. & Kumar Sahu, J. Mode area scaling with multi-trench rod-type fibers. http://apl.aip.org/resource/1/applab/v89/i11/p111119_s1. (2013). 10.1364/OE.21.001448 23389126

[34] Li M-J (2009). Limit of effective area for single-mode operation in step-index large mode area laser fibers. J. Lightwave Technol..

[35] Miluski P (2022). Large mode area fibers for single-mode transmission near 2μm Proc. SPIE.

[36] Beier F (2016). Narrow linewidth, single mode 3 kW average power from a directly diode pumped ytterbium-doped low NA fiber amplifier. Opt. Express.

[37] Samson, B., Frith, G., Carter, A. & Tankala, K. High-power large-mode area optical fibers for fiber lasers and amplifiers. *OFC/NFOEC 2008 - 2008 Conference on Optical Fiber Communication/National Fiber Optic Engineers Conference* (2008) doi:10.1109/OFC.2008.4528625.

[38] Peterka P, Dussardier B, Blanc W, Kasik I, Honzatko P (2012). Thulium-doped silica fibers with enhanced 3H4 level lifetime for fiber lasers and amplifiers. 2012 IEEE 3rd International Conference on Photonics.

[39] Kong F (2016). Large-mode-area fibers operating near singlemode regime. Opt. express.

[40] Kirchhof J, Unger S, Schwuchow A, Dellith J (2020). Optical properties of ytterbium/aluminium doped silica glasses. Opt. Mater. Express.

[41] Noronen T, Okhotnikov O, Gumenyuk R (2016). Electronically tunable thulium-holmium mode-locked fiber laser for the 1700–1800 nm wavelength band. Opt. Express.

[42] Ramírez-Martínez NJ, Núñez-Velázquez M, Sahu JK (2020). Study on the dopant concentration ratio in thulium-holmium doped silica fibers for lasing at 21μm. Opt. Express.

[43] Honzatko P, Baravets Y, Kasik I, Podrazky O (2014). Wideband thulium-holmium-doped fiber source with combined forward and backward amplified spontaneous emission at 1600–2300 nm spectral band. Opt. Lett..

